# Isolated Acromioclavicular Joint Septic Arthritis: A Case Report and Review of Literature

**DOI:** 10.7759/cureus.63590

**Published:** 2024-07-01

**Authors:** Anandu Ramanan, Vijayanand Balasubramanian, Rishab C, Palani Sami

**Affiliations:** 1 Orthopaedics, SRM Medical College, Chennai, IND

**Keywords:** acromioclavicular joint, septic arthritis, erythrocyte sedimentation rate (esr), arthrotomy, methicillin resistant staphylococcus aureus (mrsa)

## Abstract

A 60-year-old diabetic patient presented with acute pain and swelling localized to the left acromioclavicular joint. Laboratory and radiological investigations revealed the presence of pus in the left acromioclavicular joint along with bony erosion of the lateral end of the left clavicle. She was treated with open arthrotomy, debridement, and appropriate antibiotics for the causative methicillin-resistant Staphylococcus aureus (MRSA) infection. Prompt diagnosis and timely intervention can reduce the morbidity and mortality due to septic arthritis. We conducted a review of the literature on patients treated for isolated septic arthritis of the acromioclavicular joint.

## Introduction

Septic arthritis is a serious orthopedic emergency that often occurs in large joints. Even though acromioclavicular septic arthritis is uncommon, it can affect elderly individuals with compromised immune systems. Several clinical conditions, such as trauma, surgery, intra-articular injection, osteoarthritis, and intravenous drug abuse, and systemic conditions like diabetes mellitus, HIV infection, rheumatoid arthritis, and corticosteroid drug usage, can predispose to septic arthritis. Acromioclavicular septic arthritis can be misinterpreted as septic arthritis of the glenohumeral joint or degenerative disease near the latter. Existing literature on it is limited to a few case reports only.

Anatomy of the acromioclavicular joint

The acromioclavicular joint is a plane synovial joint articulation in the shoulder region between the acromion of the scapula and the lateral end of the clavicle. Two unusual features of the acromioclavicular joint are fibrocartilage lining the articular surfaces and a partially divided joint cavity caused by an articular disc. The joint capsule encloses the two articular surfaces of the acromioclavicular joint. It consists of a loose layer of fibrous tissue and an interior lining made of synovial membrane. The fibers of the trapezius muscle give support to the posterior aspect of the joint capsule. The acromioclavicular joint is supported and stabilized by three ligaments - acromioclavicular ligament, conoid ligament, and trapezoid ligament. In addition to allowing gliding motion in the superior, inferior, and anteroposterior planes, the acromioclavicular joint also has a limited degree of axial rotation.

## Case presentation

Case report

A 60-year-old lady with uncontrolled type 2 diabetes presented with left shoulder pain and swelling of a one-week duration. She had a history of fever for three days. There was no history of recent trauma. She was treated for probable rotator cuff tendinitis with interferential therapy for five days without any relief of pain. On examination, she was febrile. The left acromioclavicular joint region was swollen, tender, and warm with surrounding erythema. Shoulder abduction, forced adduction, and crossed abduction were significantly limited due to pain though rotations were painless. The total leucocyte count was 14,100/cu.mm and blood glucose measured 427 mg/dl. The inflammatory parameters ESR and CRP were 75 mm/1st hour and 113 mg/L, respectively.

**Table 1:** Laboratory investigation reports

	Patient’s value	Normal range
Total leucocyte count	14,100/microliter	4,500 – 11,000/microliter
Random blood glucose value	427 mg/dL	70 – 110 mg/dL
ESR	75 mm/hr	Less than 22 mm/hr
CRP	113 mg/dL	0 – 5 mg/dL

**Figure 1:**
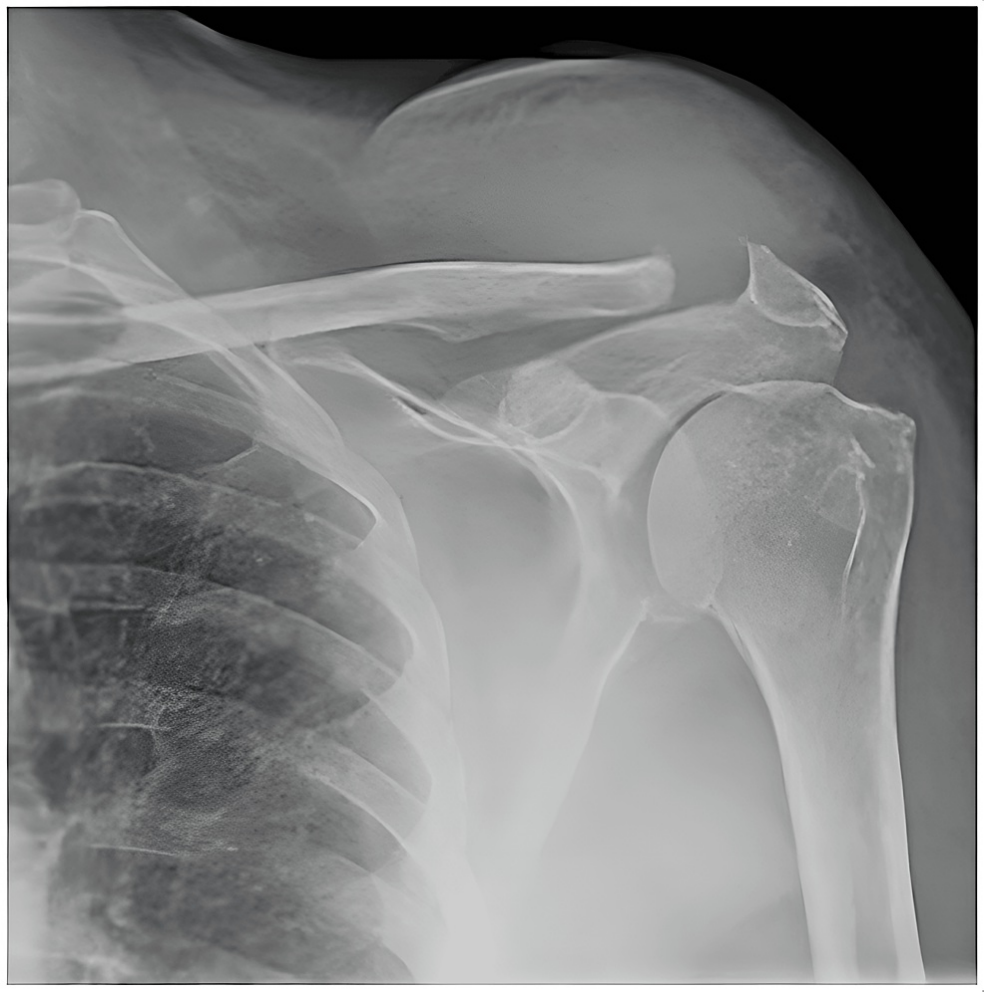
X-ray left shoulder Increased soft tissue shadow above the acromioclavicular joint and widening of the joint

Increased soft tissue shadows and erosion at the superior aspect of the left clavicle were seen in the radiograph without any sign of periosteal reaction (Figure 1).

The ultrasound scan reported mild acromioclavicular joint arthropathy with diffuse subcutaneous edema in the left supra-clavicular region with interfascial plane fluid.

**Figure 2:**
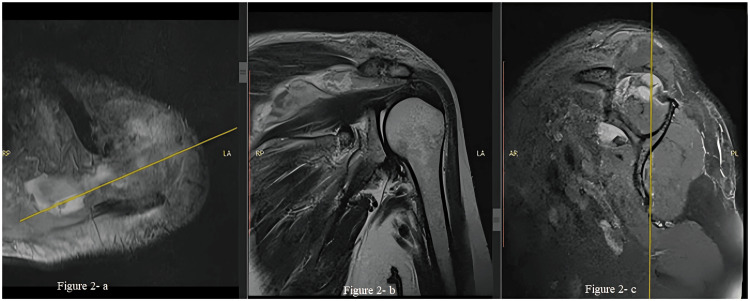
MRI left shoulder showing the fluid collection in the acromioclavicular joint (AC) tracking inferomedially 2-a) Axial cut showing abscess arising from the AC joint. 2-b) Sagittal cut showing destruction of the cartilage of the AC joint with the abscess traveling superior to the supraspinatus muscle. 2-c) Coronal cut showing the abscess arising from the AC joint superior to the supraspinatus muscle with the resultant muscle atrophy; the infraspinatus muscle was not involved.

Magnetic resonance imaging revealed irregularities in the acromioclavicular joint surface, with fluid collection measuring 8.7 x 1.4 x 1.4 cm between the supraspinatus and trapezius muscles (Figure 2). The patient was started on empiric treatment with Inj. piperacillin-tazobactam 4.5 g thrice daily after needle arthrocentesis. However, as there was no clinical improvement after 48 hours, an open arthrotomy of the AC joint and debridement were performed through an anterolateral approach.

The patient was placed in the supine position, with a sandbag under the spine and medial border of the scapula. A transverse incision from the anterolateral corner of the acromion to just lateral to the coracoid process was made. After deepening the incision through the subcutaneous fat to the deep fascia, the deltoid muscle was detached at a point well proximal to the nerve supply. An incision was made over the deep fascia along the line of the skin incision to visualize the AC joint.

The arthrocentesis and arthrotomy samples grew methicillin-resistant Staphylococcus aureus (MRSA) infection. Linezolid 600 mg intravenously twice a day was given parenterally for two weeks, followed by oral antibiotic therapy for six weeks. By 8 weeks, the inflammatory parameters showed progressive improvement, and antibiotics were discontinued. At the three-month follow-up, the patient reported no pain in the left shoulder, with a restriction of terminal 30 degrees of abduction.

Review of the literature on septic acromioclavicular arthritis

Table 2 lists the results of our review of the literature on septic acromioclavicular arthritis.

**Table 2:** Results of our literature review AC: acromioclavicular; MSSA: meticillin-sensitive Staphylococcus aureus; MRSA: methicillin-resistant Staphylococcus aureus; IVDU: intravenous drug use; WBC: white blood cell; DCE: dual channel endoscope; I&D: irrigation and debridement; ESR: erythrocyte sedimentation rate; CRP: C-reactive protein; IV: intravenous; PO: postoperative; SCM: sternocleidomastoid muscle; SC: steroclavicular; STIR: short tau inversion recovery

Author	Patient Age	Sex	Time to Presentation	Comorbidities	Side	Lab Investigation	Febrile	Imaging Studies	Bacteria (Cultured From the AC Joint)	Blood Cultures	Treatment	Outcome
Marie Bossert et al. [1]	74	M	NA	Infective endocarditis	R	WBC, CRP-elevated	Yes	NA	No organisms were identified	Positive for Staphylococcus aureus	Aspiration and two empirically selected antimicrobials were given, followed by oxacillin and gentamicin. Aortic valve replacement surgery was performed on day 16.	Died from multiorgan failure after 1 month
	55	M	NA	Dysmetabolic syndrome and gout	R	WBC, CRP-elevated	Yes	MRI	Staphylococcus aureus	Positive	Aspiration and 6 months of combined oxacillin and ciprofloxacin therapy	Healed
	64	M	NA	Chronic obstructive pulmonary disease and rheumatoid arthritis	R	WBC, CRP-elevated	No	X-ray, MRI	Negative	NA	Aspiration and 6 months of combined oxacillin and ciprofloxacin therapy	NA
	38	M	28 days	IVDU	R	WBC, CRP-elevated	Yes	X-ray, USG Scan	Negative	NA	The patient developed pain Subsequently, an abscess developed over the right acromioclavicular joint and drained to the skin. Aspiration was done and no organism was recovered from the joint aspirate. Surgery was performed to remove the abscess and to wash and debride the joint. A coagulase-negative S. aureus was recovered from the surgical specimens. A combination of two appropriate antimicrobials (rifampin and ofloxacin) was given for 8 weeks	Healed
	62	M	NA	NA	R	WBC, CRP-elevated	NA	USG scan, MRI	NA	Positive (Staphylococcus aureus)	Combination of cloxacillin and ofloxacin for 2 months	Healed
Raymond G Steinmetz et al. [2]	34	M	3 Days	Diabetes mellitus	L	WBC, ESR, CRP-elevated	Yes	X-ray, CT scan, and MRI	MRSA	Positive	I&D with DCE. IV nafcillin for six weeks	Healed. Required arthroscopic lysis of adhesions several months later
	58	M	10 Days	Diabetes mellitus, hepatitis C, IVDU	L	WBC, ESR -elevated, CRP-normal	No	X-rays normal. MRI diagnostic for septic AC arthritis, distal clavicle osteomyelitis, deltoid/supraspinatus pyomyositis	Staphylococcus aureus	Positive	Open surgical I&D with DCE. Required to repeat open I&D on postoperative day 2. IV nafcillin for 6 weeks followed by one month of trimethoprim No/sulfamethoxazole	Healed
	48	F	1 Day	Diabetes mellitus, acute myelogenous leukemia on immunosuppressants	L	WBC-normal	No	X-rays normal. MRI diagnostic for septic AC arthritis	MRSA	Positive	Aspiration and 6 weeks of IV vancomycin	Healed
Hong et al. [3]	53	M	7 Days	Hypertension	L	WBC, ESR, CRP-elevated	Yes	X-rays normal. Ultrasound and MRI diagnostic	Negative	Positive - Haemophilus parainfluenzae	Aspiration and six weeks of IV cefazolin and gentamicin	Healed
Chiang et al. [4]	55	F	3 Days	Multiple myeloma, renal insufficiency	L	WBC-normal, ESR, CRP-elevated	Yes	MRI	NA	Positive - Streptococcus pneumoniae	I&D and open AC resection and eight weeks of IV linezolid	Healed
	55 (same patient as above)	F	1 Day	Multiple myeloma, renal insufficiency	R	WBC, ESR, CRP-elevated	Yes	X-rays normal. MRI diagnostic for septic AC arthritis	NA	Positive - Streptococcus viridans	Open I&D w/DCE and six weeks of IV ceftriaxone	Healed
	79	F	4 Days	Hypertension, dementia	R	WBC-normal, ESR, CRP-elevated	No	X-rays normal.	Group B Streptococcus	NA	Aspiration and six weeks of IV ceftriaxone and Zosyn	Healed
	65	M	7 Days	Diabetes mellitus, gout, renal insufficiency	L	WBC, ESR, CRP-elevated	Yes	X-rays normal. MRI diagnostic for septic AC arthritis	Negative	Negative	Aspiration and four weeks of IV nafcillin and Zosyn	Healed
Battaglia [5]	17	M	90 Days	Nil	R	NA	No	MRI	Ochrobactrum anthropi	NA	I&D w/DCE and PO CiproþBactrim for two weeks	Healed
Blankstein et al. [6]	48	M	8 Hours	Nil	R	WBC-elevated	No	X-rays showed widening	Streptococcus viridans	Negative	I&D with open AC resection and penicillin (did not specify length)	Healed
Hammel and Kwon [7]	68	M	6 Hours	Diabetes mellitus	R	WBC, ESR-elevated	Yes	MRI diagnostic	NA	Positive - Group B Staphylococcus	6 weeks of IV ampicillin	Healed
Martinez-Morillo et al. [8]	73	M	7 Days	Cirrhosis and chronic renal failure	R	NA	No	Ultrasound	Staphylococcus aureus	Positive	I&D w/IV cloxacillin and PO ciprofloxacin for 6 weeks	Healed
	46	F	7 Days	Chemotherapy for disseminated breast cancer	R	NA	Yes	Ultrasound	Staphylococcus aureus	Positive	IV cloxacillin	Death at day 9
	72	M	10 Days	Chronic renal failure and alcoholism	L	NA	Yes	Ultrasound	Staphylococcus aureus	Positive	IV cloxacillin and PO ciprofloxacin for 6 weeks	NA
	52	M	4 Days	Chronic renal failure	NA	NA	Yes	Scan	Streptococcus pneumoniae	Positive	Penicillin G IV and amoxicillin for 8 weeks	NA
	53	M	2 Days	Diabetes mellitus	NA	NA	Yes	Scan	Streptococcus agalactiae	Positive	Penicillin G IV and PO amoxicillin for 8 weeks	NA
	71	M	5 Days	Chronic renal failure	R	NA	Yes	NA	Staphylococcus aureus	Positive	IV cloxacillin	NA
Carey et al. [9]	60	F	7 Days	Hypertension	L	WBC- normal, ESR, CRP-elevated	No	X-ray w/AC degenerative change, MRI diagnostic	Haemophilus parainfluenzae	NA	I&D and levofloxacin for 2 weeks	Healed
Cone et al. [10]	63	M	7 Days	Diabetes mellitus	L	NA	NA	Ultrasound, MRI diagnostic	Staphylococcus aureus	Positive	I&D and DCE for 2 with oxacillin for 6 to 9 weeks.	Healed
Laktasic-Zerjavic et al. [11]	44	M	6 Days	Diabetes mellitus	L	WBC- elevated	Yes	Ultrasound and Tc99 scan	Staphylococcus aureus	Positive	IV cloxacillin and gentamicin for 6 weeks	Healed
M. Martínez-Morillo et al. [12]	73	M	7 Days	Cirrhosis, chronic renal failure	R	NA	No	Ultrasound	Staphylococcus aureus	Positive	Surgical debridement, IV cloxacillin ciprofloxacin oral (6 weeks)	Healed
	46	F	7 Days	Disseminated breast neoplasia chemotherapy	R	NA	Yes	Ultrasound	Staphylococcus aureus	Positive	IV Cloxacillin	Death 9th day
	72	M	10 Days	Chronic renal failure	L	NA	Yes	Ultrasound	Staphylococcus aureus	Positive	IV cloxacillin ciprofloxacin oral (6 weeks)	Healed
	52	M	4 Days	Diabetes mellitus	NA	NA	Yes	Scan	Streptococcus pneumoniae	Positive	Penicillin G sodium intravenous Oral amoxicillin (8 weeks)	Healed
	53	M	2 Days	Chronic renal failure	NA	NA	Yes	Scan	Streptococcus agalactiae	Positive	Penicillin G sodium intravenous oral amoxicillin (8 weeks)	Healed
	71	M	5 Days	Chronic renal failure, Alcoholism	R	NA	Yes	NA	Staphylococcus aureus	Positive	IV cloxacillin	Good evolution of sepsis Death by hematoma of the rectus sheath
Saurabh Dutt et al. [13]	9	F	4 Days	Nil	L	WBC, ESR, CRP-elevated	Yes	X-rays, USG, NCCT	MRSA	NA	Aspiration followed by empirical intravenous injection of antibiotics (ceftriaxone and amikacin) was started. After the pus culture report, ceftriaxone was stopped and erythromycin was started. IV antibiotics were continued for 2 weeks followed by a period of 4 weeks of oral antibiotics	Healed
Blair Cooper et al. [14]	46	M	1 Day	NA	L	WBC, CRP-elevated	No	X-ray demonstrated minor flattening and sclerosis at the greater tuberosity suggestive of rotator cuff degenerative change	MRSA	Positive	Scope I&D and IV vancomycin for 2 weeks, switched to oral ciprofloxacin and oral clindamycin for a further two weeks.	Healed
Jija Thomas et al. [15]	64	M	14 Days	chronic obstructive pulmonary disease, rheumatoid arthritis	L	WBC, ESR, CRP-elevated	Yes	X-ray showed joint space widening with acromioclavicular joint osteoarthritis. USG shows fluid collection	Staphylococcus aureus		I&D teicoplanin followed by doxycycline for 3 weeks.	Healed
	50	F	NA	Nil	L	WBC-elevated	No	NA	NA	NA	I&D started with IV vancomycin and IV piperacillin-tazobactam, changed to oxacillin and I&D was repeated after 2 days due to increasing pain and swelling of the SCM muscle, an MR scan revealed fluid collection on STIR images in the SC joint, edema of the proximal bone, and liquefaction of the entire SCM muscle from the SC joint to the base of the skull, then 2 weeks of oxacillin IV followed by 4 weeks of outpatient ceftriaxone IV 2 g per day.	Healed
Mark Williams [16]	69	M	4 Days	Metabolic syndrome (hypertension, hypercholesterolemia, obesity, and impaired glucose tolerance), Klinefelter’s syndrome, and mild renal insufficiency.	L	WBC, ESR, CRP-elevated	Yes	X-ray - normal	Staphylococcus aureus	Positive	Arthroscopic washout, post-washout, and intravenous flucloxacillin (2 g, four times a day) were started empirically. IV clindamycin (900 mg, four times a day) was started for 6 days.	Healed
Adam Oswald et al. [17]	51	M	3 Days	Diabetes mellitus, schizophrenia, and IVDU	R	WBC, ESR, CRP-elevated	No	X-ray showed mild acromioclavicular arthropathy. CT revealed fat stranding consistent with cellulitis but no focal fluid collection or aggressive bone lesions; MRI	NA	Streptococcus agalactiae group B	Start on IV vancomycin and ceftriaxone. He underwent transthoracic echocardiography to evaluate for endocarditis, which was negative for any significant findings or vegetation	NA

## Discussion

Acromioclavicular joint septic arthritis is a rare entity that can affect elderly individuals with compromised immune systems. Usually, acromioclavicular joint septic arthritis co-exists with septic arthritis of the shoulder joint, so the actual incidence of AC joint septic arthritis alone is unknown [18]. The patients usually present with pain over the shoulder with overhead activity or with cross-body arm adduction. On examination, patients have pain on direct palpation of the AC joint.

Only a few case reports have been reported so far in the English literature. Patients with septic AC arthritis frequently present later than expected because of misdiagnosis as pyomyositis or underdiagnosis. The patients' ages ranged from 9 to 79 years old, with a mean age of 52.66 years. Among those patients, 26 were over 50, and 11 were under 50. The duration of the presentation varied from six hours to three months. Of the cases, 75.6% (28 out of 37) were male. Of the 23 cases, 13 were on the right side and 10 were on the left. Twenty-one out of the 35 cases (55.2%) occurred in individuals with weakened immune systems like diabetes mellitus. Three out of 37 cases were intravenous (IV) drug abusers. Other predisposing factors described were HIV, renal failure, cirrhosis, lymphomas, myeloma, rheumatoid arthritis, and cytotoxic chemotherapy.

Staphylococcus aureus was the most frequently detected pathogen followed by streptococcus pneumoniae, especially in hematologic malignancies. MRSA (4), Streptococcus pneumonia(2), Streptococcusagalactiae (2), Streptococcus viridians (1), Group B streptococcus(1), Ochrobactrumanthropi, and Haemophilusinfluenzae were among the other organisms described as causative agents in literature. Most of these patients show positive blood cultures indicating a hematogenous spread.

Since the AC joint is a smaller joint, septic arthritis can be very damaging. To avoid morbidity, a high degree of suspicion must be raised for an accurate and prompt diagnosis. It can be difficult to differentiate painful limited shoulder motion in these patients from glenohumeral involvement because they often have limited shoulder motion in both active and passive motion. The location of the anterior shoulder pain and the boggy feeling over the acromioclavicular joint, which is caused by surrounding pyomyositis, suggest the diagnosis.

In 82.6% (19 out of 23) of the cases, leucocytosis was present. Inflammatory parameters were elevated in 50% of cases. X-rays have revealed an enlargement of the AC joint and deterioration of the surrounding bone. Ultrasound can guide joint aspiration and can detect joint effusion in the AC joint. Magnetic resonance imaging (MRI) and ultrasound offer a more accurate and sensitive diagnosis early on than conventional X-rays. MRI and ultrasound allow us to make an earlier diagnosis and evaluate the regional spread of the infection. Greater tissue definition, early damage detection, sensitivity, specificity, and operator independence are all features of magnetic resonance imaging [12]. It reveals thickening of the joint capsule, synovium, and apparent expansion of the joint space. So, MRI is crucial for early diagnosis, detecting osteomyelitis, assessing rotator cuff integrity, and assessing glenohumeral joint involvement [15].

Since arthrocentesis offers quick confirmation and directs treatment, it is the gold standard for diagnosing AC septic arthritis. Joint aspiration followed by saline infiltration may be used to know the shoulder joint extension. However, the bony anatomy of the joint may make it technically difficult to obtain a substantial aspirate for microbiological diagnosis [15]. Once a microbiological diagnosis is made, appropriate antibiotic therapy needs to be selected.

Treatments for septic arthritis of the AC joint that have been demonstrated to be successful include aspiration of the joint, joint irrigation, surgical debridement, and resection of the lateral end of the clavicle or AC joint along with an IV or oral antibiotic course. Adequate drainage of the joint and administration of antibiotics is the mainstay treatment for acromioclavicular joint septic arthritis [19]. The minimum duration of antibiotic use should not be less than four weeks [20]. Surgical debridement, whether it be open or arthroscopic, provides the most comprehensive debridement of any associated pyomyositis and subacromial or subdeltoid extension in addition to the AC joint. Conventionally distal clavicle excision is suggested during debridement of acromioclavicular joint septic arthritis [21,22]. Out of these patients, 41.6% had surgical debridement, 37.8 % had medical management, and 21.6% had aspiration followed by antibiotic treatments. Adams and McDonald reported a patient with sarcoidosis and cryptococcal arthritis of the AC joint that was treated with irrigation and debridement, distal clavicle excision, and IV antibiotics [23]. They did a resection of the whole AC joint instead of a simple resection of the distal clavicle. On average, six weeks of appropriate antibiotics provide successful clinical results.

## Conclusions

Acromioclavicular joint septic arthritis is a rare condition that occurs in individuals with compromised immune systems. Various immunodeficient conditions predispose a patient to septic arthritis of the acromioclavicular joint. For an early diagnosis, a thorough clinical assessment of the AC joint is necessary. The diagnosis is confirmed via a combination of laboratory test findings and modern imaging investigations. Aspiration of the AC joint, joint irrigation, surgical debridement, and resection of the lateral end of the clavicle or AC joint combined with an IV or oral antibiotic course are effective treatments for septic arthritis of the acromioclavicular joint septic arthritis. Timely diagnosis and treatment with appropriate antibiotics are essential to prevent morbidity and sepsis.
